# Oleaginous yeast, *Rhodotorula paludigena* CM33, platform for bio-oil and biochar productions via fast pyrolysis

**DOI:** 10.1186/s13068-023-02270-x

**Published:** 2023-02-05

**Authors:** Pongsatorn Poopisut, Pasama Boonyanan, Pailin Boontawan, Ekarong Sukjit, Nuttapan Promsampao, Nuwong Chollacoop, Mariena Ketudat-Cairns, Adisak Pattiya, Apichat Boontawan

**Affiliations:** 1 School of Biotechnology, Institute of Agricultural Technology, Suranaree University of Technology, 111 University Avenue, Muang District, Nakhon Ratchasima, 30000 Thailand; 2 Center of Excellent in Agricultural Product Innovation, Suranaree University of Technology, 111 University Avenue, Muang District, Nakhon Ratchasima, 30000 Thailand; 3 School of Mechanical Engineering, Institute of Engineering, Suranaree University of Technology, 111 University Avenue, Muang District, Nakhon Ratchasima, 30000 Thailand; 4 Biomass Pyrolysis Frontier Research Group, Faculty of Engineering, Mahasarakham University, Kamriang, Kantharawichai, Maha Sarakham 44150 Thailand; 5 National Energy Technology Center, 114 Thailand Science Park, Phahonyothin Rd., Khlong Nueng, Khlong Luang, Pathum Thani 12120 Thailand

**Keywords:** Biochar, Bio-oil, Oleaginous yeast, Pyrolysis, *Rhodotorula paludigena* CM33, Simulated distillation

## Abstract

An oleaginous yeast *Rhodotorula paludigena* CM33 was pyrolyzed for the first time to produce bio-oil and biochar applying a bench-scale reactor. The strain possessed a high lipid content with the main fatty acids similar to vegetable oils. Prior to pyrolysis, the yeast was dehydrated using a spray dryer. Pyrolysis temperatures in the range of 400–600 °C were explored in order to obtain the optimal condition for bio-oil and biochar production. The result showed that a maximum bio-oil yield of 60% was achieved at 550 °C. Simulated distillation gas chromatography showed that the bio-oil contained 2.6% heavy naphtha, 20.7% kerosene, 24.3% biodiesel, and 52.4% fuel oil. Moreover, a short path distillation technique was attempted in order to further purify the bio-oil. The biochar was also characterized for its properties. The consequence of this work could pave a way for the sustainable production of solid and liquid biofuel products from the oleaginous yeast.

## Introduction

Numerous efforts are underway to find alternative resources and technologies to replace the depleting fossil fuels. Thermochemical conversion of biomass, such as pyrolysis, is one of the promising techniques for bioenergy production [1]. Pyrolysis refers to thermal degradation of organic material by cracking the chemical bonds in an inert or non-oxidative atmosphere. It is recognized as a flexible sustainable technology because the products can be obtained in the form of gas (syngas), liquid (bio-oil), and solid (biochar), which can be utilized in the renewable energy and other applications [2].

In pyrolysis, biomass is ground before being fed into a reactor in the absence of oxygen. The chemical structure of biomass is broken down into small molecules under high temperature typically in the range of 400–600 °C [3]. The pyrolysis vapor can be condensed by a condenser unit, and the dark brown liquid formed is called bio-oil or pyrolysis liquid. In addition, the solid biochar is also produced and can be separated by a cyclone unit [4]. Numerous sources of lignocellulosic biomass were investigated by pyrolysis technique, such as corn cob [5–7], water hyacinth [8, 9], woods [10–13], sugar cane bagasse [14–16], wheat straw [17, 18], and cassava rhizome [19, 20]. However, one of the main drawbacks of the bio-oil produced from these resources is that their composition is very similar to that of the original biomass and is very different from petroleum derived fuels and chemicals. The bio-oil tends to have high water and oxygen contents, leading to a lower gross calorific value and poor stability [21, 22].

Pyrolysis of triglyceride-based biomass has been introduced in order to improve the fuel properties especially in an increase in the olefin, and aromatic content [23, 24]. Several vegetable oils, animal fat, and some other similar sources have been investigated; however, an extensive use of edible oils for bio-oil production may lead to food crisis [25]. These problems can be solved by using sustainable feedstocks particularly in the form of microbial oil, such as microalgae [26–29] and yeast [30–33]. There are several advantages of microorganisms, especially oleaginous yeast to be used as a feedstock. For example, the oleaginous yeasts generally contain high proportion of lipid, have high growth rate, and are easy to cultivate and harvest. Therefore, oleaginous yeast has been considered as potential feedstock for thermochemical conversion, such as pyrolysis process, for production of biofuels. Examples of previous studies investigated oleaginous yeast in comparison with some biomass species are summarized in Table 1. It can be seen that most the optimum pyrolysis temperature for maximizing the bio-oil yield is around 500 °C and the bio-oil yield ranges from 42 to 73 wt% depending on feedstock. Studies on pyrolysis of typical woody and agricultural biomass are widely available. However, pyrolysis of oleaginous yeast is very limited. In addition, to the best of our knowledge, pyrolysis of oleaginous yeast *R. paludigena* CM33 has not been investigated.

**Table 1:** Previous studies on pyrolysis of oleaginous yeast in comparison with other biomass types

Biomass	Reactor type	Optimum pyrolysis temperature (°C)	Product yield (wt%)			Refs.
			Bio-oil	Biochar	Gas	
Oleaginous yeast (*Trichosporon fermentans* biomass)	Fixed bed	500	42	18	40	[32]
Eucalyptus wood	Fluidized bed	500	53	14	33	[34]
Cassava rhizome	Fluidized bed	472	63	25	12	[35]
Contaminated sawdust	Circulating fluidized bed	500	67	18	15	[36]
Palm shell	Helical screw fluidized bed	500	73	12	15	[37]
Jatropha oil cake	Fluidized bed	500	64	4	32	[38]

In 2020 and 2021, small-scale fixed-bed pyrolysis experiments were carried out applying 0.5–1.0 g of *Trichosporon fermentans* yeast cultivated by refined soybean oil wastewater [32, 39]. The yeast samples were heated at a rate of 10 °C/min in a tube furnace from ambient temperature to 300–500 °C with 30 min holding time. The evolved pyrolysis vapor was condensed to bio-oil. The bio-oil yield was dependent on the final pyrolysis temperature. At 500 °C, the maximum yeast bio-oil yield of 42% was obtained [32]. The bio-oil was subsequently analyzed by GC-TOF/MS technique. It has been shown that the yeast bio-oil was a mixture of low-to-intermediate molecular weight (C7–C18) compounds representing saturated alkanes as well as aromatic and oxygenated compounds [39]. The bio-oil hydrocarbon contents were 28% when applying the pyrolysis temperature of 500 °C and became 41% when the temperature was increased to 600 °C [32].

Although pyrolysis of oleaginous yeasts has been recently investigated, the insight into the characteristics of the yeast-derived bio-oil is still limited as only GC-TOF/MS method was applied due to the very small quantity of the produced bio-oil. Not only that, to the best of the authors’ knowledge, there is a conspicuous lack of research work on characteristics of another important secondary product, biochar derived from pyrolysis oleaginous yeast. Therefore, this study aims to fill this knowledge gap by performing pyrolysis of oleaginous yeast in a larger scale in order to produce enough amounts for bio-oil and biochar for characterization. The novelty of the present study lies in the production and characterization of bio-oil and biochar from pyrolysis of an oleaginous yeast *R. paludigena* CM33.

In this work, the effect of pyrolysis temperature in the range of 400–600 °C was examined to produce bio-oil and biochar. The bio-oil was analyzed for heating value, chemical composition using GC/MS, short path distillation, simulated distillation, and lubricity. The solid char was subjected to proximate, ultimate, and heating value analyses; surface characteristics, including surface area, porosity, surface morphology by SEM, and crystallographic structure by XRD; and chemical functional group by FTIR.

## Materials and methods

### The yeast strain and feedstock preparation

*Rhodotorula paludigena* CM33 was one of the oleaginous yeasts isolated from various natural habitats by our laboratory [40]. The strain was kept at − 80 °C in yeast extract peptone dextrose (YPD) media with 20% (% *v*/*v*) glycerol containing (g/L) of; 20 g glucose, 10 g yeast extract, and 20 g peptone, respectively. The seed culture was prepared in a 5-L YPD medium, and the batch fermentation was performed in a pre-pilot-scale 50-L bioreactor with the working volume of 35-L (BE Marubishi, Thailand). For comparison purpose, the fermentation broth in this study was prepared using 80 g/L of 4 main carbon sources, including glucose, hydrolyzed starch, glycerol, and sugar cane molasses, respectively. Other nutrients contained (g/L) of 0.75 g yeast extract, 0.55 g (NH_4_)_2_SO_4_, 0.4 g KH_2_PO_4_, and 2.0 g MgSO_4_.7H_2_O, respectively. During fermentation, temperature was maintained at 30 °C under the aeration rate of 0.75 VVM, and the agitation rate of 150 rpm. For cell recovery, the cross-flow microfiltration experiment was employed with the pore size of 0.1 µm (Synder Filtration, USA) using concentration mode [41]. When the cell concentration reached approximately 200 g/L, the cell was washed by an addition of tap water at the same rate of the permeation rate. The washing step finished when the color of the permeate had no yellowish color. Subsequently, the yeast was dehydrated by using a spray dryer (Buchi mini spray dryer B-290, Germany). Finally, the dried yeast powder was subjected to be used for the pyrolysis experiments.

### Pyrolysis experiments

**Fig. 1:**
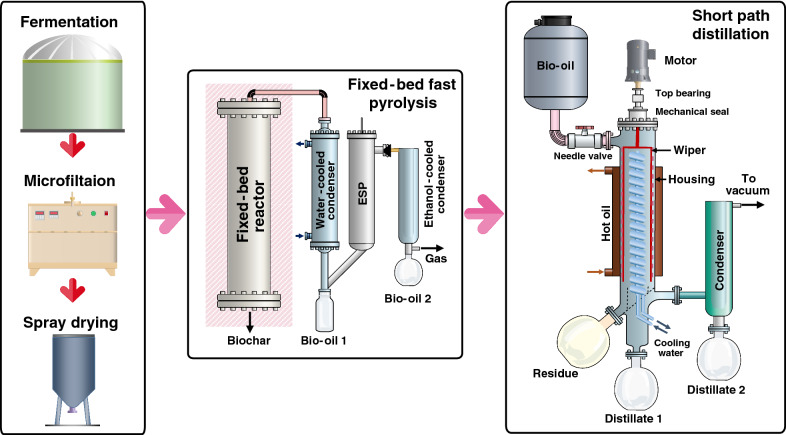
Schematic diagrams of a fixed-bed fast pyrolysis reactor and a short path distillation apparatus

The sprayed-dried oleaginous yeast powder was directly fed into a pyrolysis reactor as illustrated in Fig. 1. Briefly, the pyrolysis reactor was made of a 304 stainless-steel pipe with an inside diameter of 42 mm and a height of 450 mm. It was modified from our previous work [4]. The reactor was installed in a tailor-made muffle furnace of which the temperature was controlled by a PID mode. The reactor was operated at temperatures between 400 and 600 °C, respectively. Approximately 100 g of yeast powder was placed in the reactor and heated to the temperature setpoint. The produced pyrolysis vapor was quenched by an ethanol-cooled condenser at − 10 °C, and the aerosols were trapped by an electrostatic precipitator (ESP) at operated at 15 kV. After the process, the biochar was finally removed out from the reactor for further characterization.

### Short path distillation (SPD)

Short path distillation is a technique that include the distillate traveling in a short distance. By applying an extremely low pressure, it is very useful to purify high boiling point bioproducts ranging from fish oils to biodiesel [42, 43]. In this work, the crude bio-oil was heated at 70 °C before it was fed to the SPD unit (LAB 1st MD-150, China) with the help of a needle valve as shown in Fig. 1. The rotating wiper is responsible for the formation of a thin film on the internal heating surface. The SPD had a length between the internal wall and the condenser located at the middle of the column, and the distillate product can be collected in a glass reservoir (distillate 1). The residue or stillage was collected in a residue reservoir. The distillation temperature was investigated between 150 and 200 °C, vacuum pressure was set at 10 Pa, and temperature of a condenser inside the column was maintained at 50 °C by recirculating the cooling water. The crude bio-oil was fed at 400 mL/h. In addition, another condenser was set at approximately − 10 °C in order to condense low boiling point hydrocarbons as shown for the distillate 2.

### Analysis

#### Lipid content

The total lipid content of the yeast cell was determined by an extraction using petroleum ether as the organic solvent. After filtration, the lipid residue was weighed after solvent evaporation. The fatty acid characterization was analyzed through fatty acid methyl ester (FAME) by transesterification with methanol. Briefly, 25 mg of the extracted yeast oil was placed in a test tube for mixing with 0.5 M methanolic NaOH 1.5 mL. Then, oxygen was removed by feeding of nitrogen gas and was heated for approximately 2 min at temperature 80–90 °C. Subsequently, it was left to cool, and 2 mL BF_3_ solution was added. The mixture was further heated for 30 min and was shaken periodically. Then, it was mixed with 1 mL of iso-octane and 5 mL of 36% NaCl solution. After phase separation, the upper layer was collected, and the raffinate was extracted again with the same amount of iso-octane. The sample of 1 µL was injected to a GC (Agilent 7890A, USA) equipped with a FID detector. The GC column was Agilent’s J&W CP*-*Sil 88 (100 m × 0.25 mm × 0.2 µm) which provided a great efficiency and resolution for FAME compounds. The injector temperature was 240 °C, split 50:1, and the detector temperature was 250 °C, respectively.

#### Bio-oil analysis

Compositional analysis and quantification of the detected compounds within the obtained bio-oil were performed by a GC–MS analysis (Agilent 7890A GC with Agilent 7000 Triple Quad GC/MS/MS, USA). Between 0.20 and 0.25 g of the pyrolysis liquid (either aqueous, either organic phase) was mixed with 100 μL of a 2.5 wt% fluoranthene (98%, Sigma Aldrich) solution in acetonitrile (99.5%, Carl Roth) as internal standard and diluted with 5 g acetonitrile. This solution with ca. 5 wt% analyte was injected (injector temperature of 250 °C, split ratio of 1:100), and was separated on an RTX-1701 chromatographic column (Restek, 60 m × 0.25 mm, 0.25 µm). Measurement for the energy of combustion was carried out by using a bomb colorimeter (IKA, C5000, Germany). Water content of the sample was measured by a Karl Fischer titration method. Simulated Distillation Gas Chromatography (SDGC) is a GC method that combine with simulation distillation software used to characterize crude oil sample. This technique was used to measure boiling range distribution. The bio-oils were analyzed for boiling range distribution measurement of Simulated Distillation of ASTM D 2887. The model of Simulated Distillation Gas Chromatography is Varian CP-3800 (USA) that was used in this experiment.

Lubricating properties of bio-oil derived from oleaginous yeast were carried out on a high frequency reciprocating rig (HFRR, United Kingdom) and the test parameters were set according to the test methods specified in ASTM D 6079. Experiments were repeated three times and repeatability was demonstrated to be less than 20 µm, which is acceptable according to the ASTM standard with a repeatability of 50 µm and reproducibility of 80 µm. To evaluate the lubricity of the bio-oil, an average wear scar diameter on the ball specimens was measured by the optical microscope with 100 × magnification. Microscopic observation of wear scar on disk specimens was made in a field emission scanning electron microscope (Zeiss Auriga, Germany) equipped with energy-dispersive X-ray spectrometry (EDX).

#### Biochar analysis

Proximate analysis was carried out to determine percentages of moisture, volatile matter, fixed carbon, and ash. It was done by following the ASTM E1756 for moisture content, ASTM E872-82 for volatile matter content, and ASTM E1755 for ash content, whereas the fixed carbon was calculated by difference. Carbon, Hydrogen, and Nitrogen (CHN) analyzer was used to measure carbon, hydrogen, and nitrogen elemental content in samples. The biochar was mashed and sieved to be the size of about 1 mm, and around 0.2 g was used for CHN analysis. The instrument utilizes a combustion technique and provides a result within 4.5 min for all the elements being determined. The sample was weighed and encapsulated. The final results were typically displayed in weight percent or parts-per-million.

Thermogravimetric analysis (TGA) is a method of thermal analysis used to examine the weight change that occur as the sample was heated at constant rate. The dried yeast and biochar were measured percent of moisture, volatile matter, ash, and fixed carbon in the sample by monitoring the weight change. The TGA model of LECO 701 was used to determine biochar’s thermal stability.

X-Ray Diffractometer (XRD) is one of the microstructural analytical techniques that study the crystal structure. The sample was identified the crystalline phases present in a material, and thereby reveal chemical composition information. The XRD (BRUKER D2 PHASER XRD) was used to investigate biochars; it was operated at the step time of 0.5 and 2θ of range 10–80°.

Fourier Transform Infrared Spectroscopy (FTIR) was employed to measure the absorption of infrared radiation by the sample material versus wavelength. The infrared absorption bands identify molecular components and structures. The obtained biochar was analyzed functional group by using a FTIR (BRUKER, TENSOR 27-Hyperion, Germany).

The specific surface area of a sample was determined by BET related to total surface area, porous structure, and pore size distribution. This information was used to predict product characteristics. Furthermore, it is useful in evaluation of product performance and manufacturing consistency. Determination of the specific surface area (m^2^/g) of biochar samples was investigated by a BET instrument (Micromeritics ASAP 2020 plus, USA). The samples were dried with nitrogen purging or in a vacuum applying elevated temperatures. The volume of gas adsorbed to the surface of the particles. The amount of adsorbed gas was correlated to the total surface area of the particles, including pores in the surface. The calculation was based on the BET theory. Traditionally, nitrogen is used as adsorbate gas. Gas adsorption also enables the determination of size and volume distribution of micropores (0.35–2.0 nm).

## Result and discussion

### Fermentation and biomass characterization

**Fig. 2:**
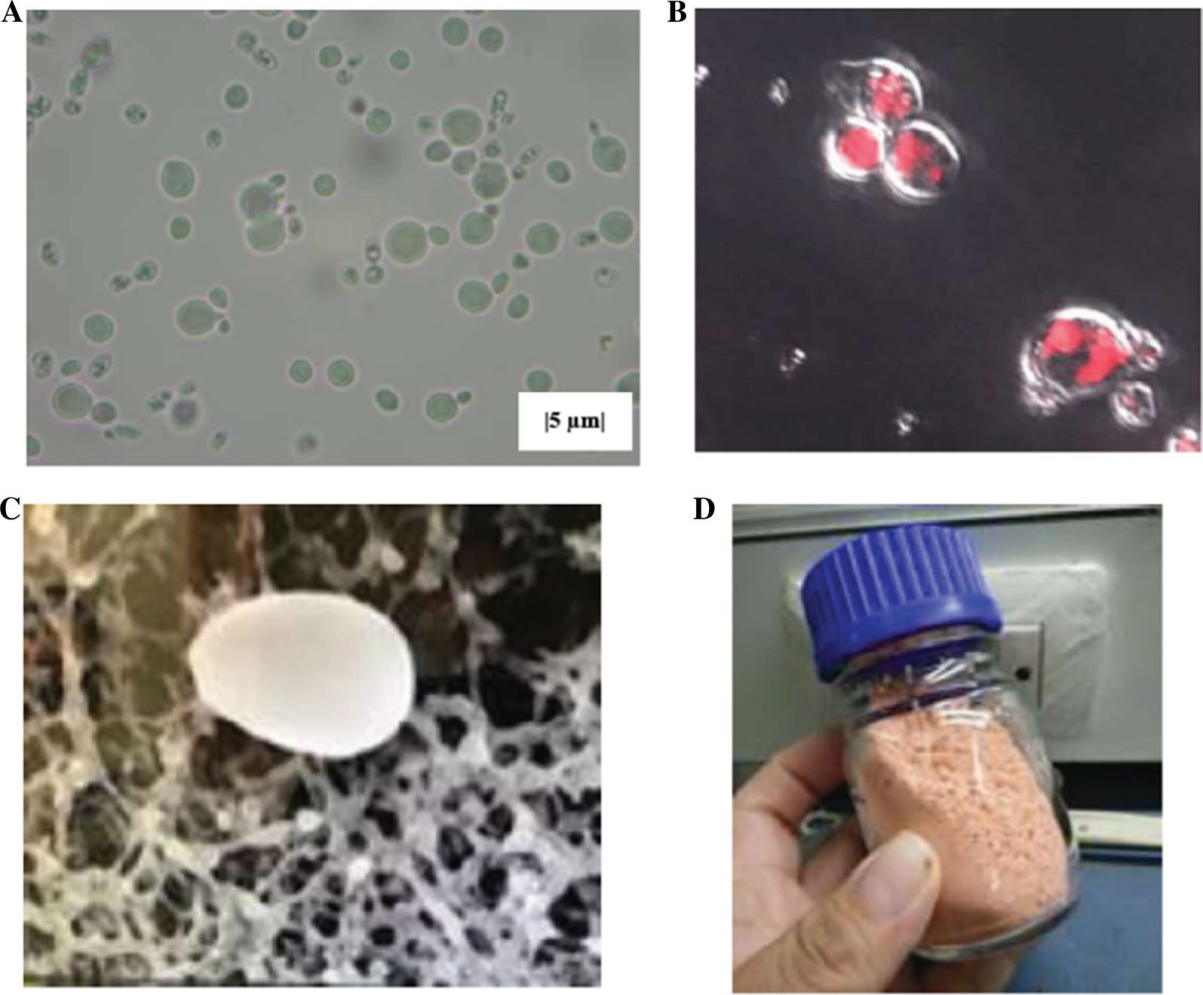
Cell of *R. paludigena* under light microscope (**A**), fluorescens microscope showing the lipid vesicle stained with Nile Red (**B**), scanning electron microscope (**C**), and the spray dried yeast powder prior to pyrolysis (**D**)

Figure 2A illustrates the yeast cells under light microscope showing that the red pigment was accumulated on the outer cell membrane. In addition, the lipid detection with Nile Red staining was carried out by using a fluorimetry method [44]. Lipid droplets inside the cells were observed under a fluorescence microscope (Olympus BX61) equipped with a fluorescence emitter using a 460–490 nm excitation filter, a 500 nm Dichromatic Mirror, and a 520 nm barrier filter (U-MWB2). Figure 2B shows the lipid vesicle observed inside the cell. In addition, typical oval shape of the cell with the size of approximately 5 µm was shown by the SEM image in Fig. 2C. Being unicellular, it has advantage over the conventional processing of other plant biomasses since it does not require the energy-consuming grinding step. In addition, the cell can be simply dried into powder by using a spray dryer as shown in Fig. 2D.

**Fig. 3:**
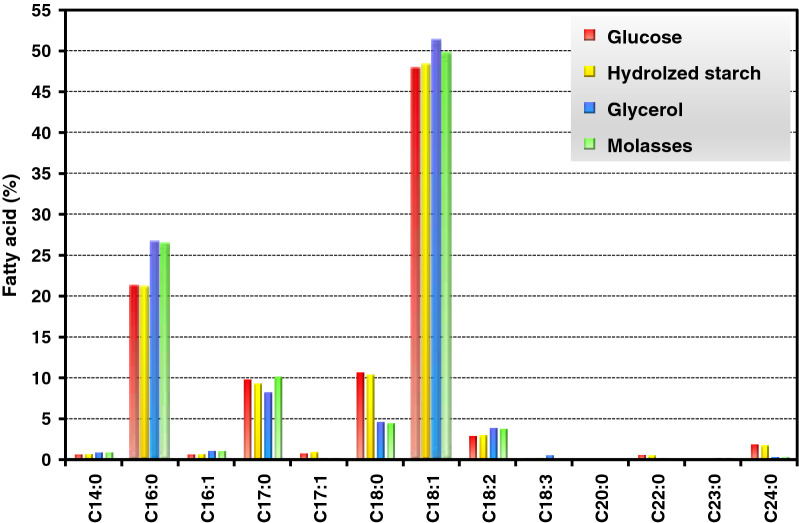
Fatty acid profiles of *R. paludigena* CM33 grown on different carbon sources

Oleaginous yeast synthesizes neutral lipids in the form of monoacylglycerols (MAGs), diacylglycerols (DAGs), and triacylglycerols (TAGs) in their lipid bodies. Culture conditions, such as temperature, pH, C/N ratio, and oxygen, played an important role on the characteristic of lipid accumulation in the cell [25]. However, investigation of these parameters was not the scope of this work. The lipid profile of the *R. paludigena* CM33 grown on different carbon sources is shown in Fig. 3. Experimental result showed that the lipid contents of the yeast grown on glucose, hydrolyzed starch, glycerol, and sugarcane molasses were measured at 27.32, 27.18, 19.55, and 21.33 wt%, respectively. In general, the yeast possesses ability to synthesize C14:0 (myristic acid), C16:0 (palmitic acid), C18:0 (stearic acid), C18:1(oleic acid), C18:2 (linoleic acid), C18:3 (linolenic acid), C20:0 (eicosanoic acid), and C24:0 tetracosanoic acid, a long-chain fatty acid, respectively. For all carbon sources, the oleic acid appears as the highest compositions of all fatty acid. Interestingly, odd-chain fatty acids were also detected especially heptadecanoic acid (C17:0) and cis-10-heptadecenoic acid (C17:1), respectively. In addition, the dark red color of the extracted oil suggests that it is a good source of carotenoids which can also be applied in food and bio-pharma industries [45]. The high lipid content of the biomass suggested that it was an ideal feedstock for biofuel production. However, nowadays glycerol is becoming one of the preferred fermentation substrates due to the thriving biodiesel industry, and agro-industry wastes especially glycerol could be the appropriate choices for further process development as they are not competing with human diet. Thus, in this research glycerol was used as substrate for the culture of oleaginous yeast.

### Pyrolysis product distribution

**Fig. 4:**
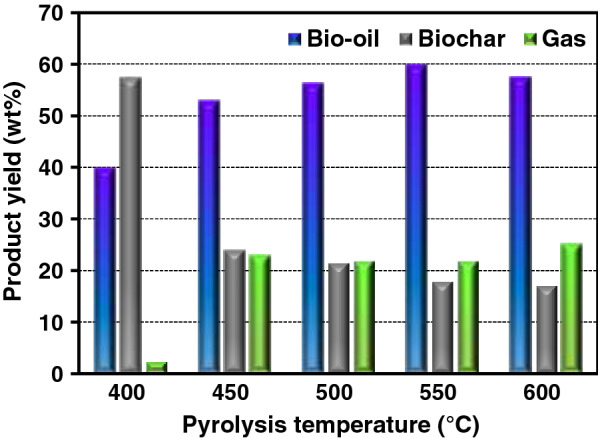
Percent yield of bio-oil, biochar, and gas at different temperatures

In pyrolysis, biomass decomposed to condensable, non-condensable vapors, as well as biochar. In this work, the condensable vapor was condensed into bio-oil, while the gas left the system without any analysis. By controlling the operating temperature, the yield of each component can then be analyzed. Figure 4 shows the percent yield of bio-oil, biochar, and gas at different operating temperatures. The yield of gas was calculated by the difference of initial biomass and product yields. It was evident that pyrolysis temperature greatly affected the pyrolysis product distribution. The bio-oil yield constantly increased with an increasing temperature from 400 to 550 °C reaching the maximum yield of 60 wt%. This increase is related to the decrease of biochar yield from 57.5 to 17.1 wt%. This is due to the enhanced devolatilization of the oleaginous yeast upon heating. However, the bio-oil yield reduced to 57.7 wt% at 600 °C. The consequence was because the primary thermal degradation of biomass occurred at lower temperature. After that, subsequent increased in the temperature further crack the volatile materials into low-molecular weight organic compounds and gases rather than biochar [12]. The highest biochar yield of this work was 57.5% at 400 °C. The biochar yield could be increased more if the pyrolysis temperature is lowered than 400 °C. However, the increase yield of biochar could come with the residual lipid since it is known from a previous study that the decomposition of lipids in oleaginous yeast occurred around 392–516 °C [39]. Therefore, lower temperatures than 400 °C would produce biochar contaminated with oleaginous yeast lipids or volatiles. In a large-scale continuous pyrolysis system, small particles of biochar may be entrained out of the reactor chamber and contaminate the bio-oil product. Therefore, it is suggested that a pyrolysis unit could install a hot vapor filtration to remove the biochar fines from the liquid product using a moving-bed granular filter [46].

The gas yield increased significantly when increasing temperature from 400 to 450 °C after which slight changes occurred. Further increase of the temperature to 600 °C led to a slight reduction of bio-oil yield. This reduction corresponds to the slight increase of non-condensable gas yield. Therefore, it is likely that at the pyrolysis temperature higher than 550 °C, secondary thermal cracking of the oleaginous yeast volatile initiated leading to the production of permanent gas. At the pyrolysis temperature of 550 °C where the maximum bio-oil yield was achieved, the gas contained 52.4% combustible gases, including 17.3% methane, 9.8% ethane, 1.7% ethylene, 8% propane, 11.4% butane, and 4.2% carbon monoxide, and 47.6% non-combustible carbon dioxide. This shows that it is possible to exploit the gaseous product as bio-fuel since it contains combustible gases with heating value of around 35.9 MJ/m^3^, which is similar to that of natural gas.

The maximum bio-oil yield of azolla, brown seaweed, and water hyacinth were reported at 38.5, 43.4, and 24.6 wt%, respectively [9]. Moreover, triglyceride material was investigated for pyrolysis process, such as the soybean oil with different amounts of double bonds in the chain. The yield was reported around 61–68% at 525 °C, and increased with higher amounts of hydrogenated fat or saturated fatty acid in the sample [23]. In this work, the maximum bio-oil yield from dried *R. paludigena* CM33 was 60 wt% at 550 °C. The different physical and chemical properties of raw materials affected significantly to yield and characteristic of bio-oil by decomposition and multiple complex reaction. In addition, the bio-oil yield may be reduced because some products are decomposed into non-condensable gas or cannot capture condensate gas. It is known from previous studies [32, 47, 48] that at the pyrolysis temperature of 500 °C or higher, the decomposition of lipids in the oleaginous yeast occurred leading to the conversion into bio-oil and non-condensable gases.

### Bio-oil characteristics

#### High heating value

The high heating value (HHV) of bio-oil was measured by bomb calorimeter as shown in Table 2. It was found that the bio-oil HHV was approximately 36 MJ/kg and was hardly influenced by pyrolysis temperature. The typical HHVs of crude oil and biodiesel are 45.5 and 40.2 MJ/kg, respectively [5]. In comparison with crude oil, bio-oil from oleaginous yeast had slightly lower HHV due to the presence of water and oxygenates. The HHV of distillated product was nearly the same as that of biodiesel.

**Table 2:** High heating value (MJ/kg) of bio-oil produced at different temperatures

Bio-oil	HHV (MJ/kg)
400 °C	–
450 °C	36.4
500 °C	36.1
550 °C	35.9
600 °C	35.7
Distilled fraction (SPD)	37.5
Stillage	34.9

#### GC/MS

**Fig. 5:**
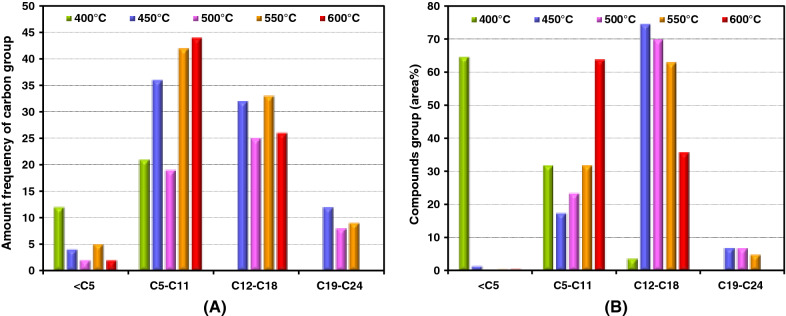
Distribution of compounds in bio-oils according to carbon number **A** amount of frequency of carbon group and **B** compounds group by carbon in chain (%V)

According to the GC/MS analysis, the compounds group and amount of frequency of carbon group in the bio-oils according to the number of carbon atoms in the chain are shown in Fig. 5. The most frequency of compounds was in the range of C5 to C11 as shown in Fig. 5A. The volume of compound groups composed range of C16 to C18, and light hydrocarbon (< C8) is shown in Fig. 5B. The main fatty acid in oleaginous yeast was decomposed into randomly shorten-chain hydrocarbon. Although the cracking fatty acid increased with rising of temperature, the bio-oil yield might be decreased because when some product was broken down into non-condensable gas or condensable gas could not be trapped.

#### Short path distillation

**Fig. 6:**
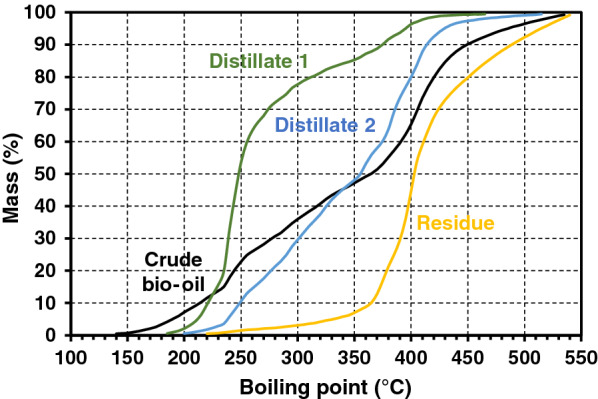
Simulated distillation curve of crude bio-oil, distillate 1, distillate 2, and residue

**Fig. 7:**
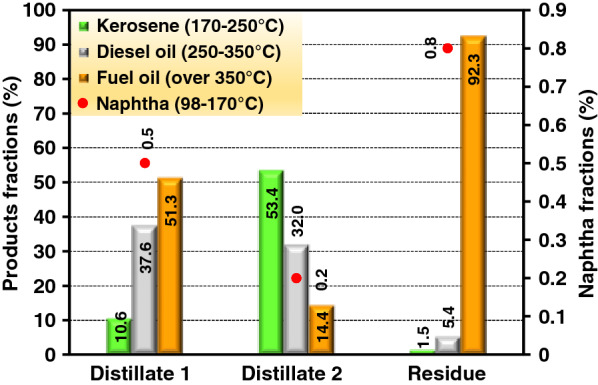
Percent content of each distillate products by short path distillation (SPD)

The content of crude bio-oil from optimal pyrolysis process at 550 °C was analyzed by simulated distillation gas chromatography (SDGC). The fuel can be distilled into heavy naphtha (98–170 °C), kerosene (170–250 °C), diesel (250–350 °C), and fuel oil (> 350 °C). The distillation curve of crude bio-oil is shown in Fig. 6. The content of crude bio-oil is presented in Fig. 7. It can be seen that the bio-oil contains 2.6% heavy naphtha, 20.7% kerosene, 24.3% diesel, and 52.4% fuel oil. The heavy naphtha content was very low because it may not be trapped by the condenser and ESP operated at room temperature. The short path distillation apparatus was previously used to separate bio-oil from fast pyrolysis of sawdust at temperature 550 °C, giving a maximum distillates yield of 85% at 130 °C and 60 Pa [43]. In this work, the distillation curves of the distillates from short path distillation (SPD) at 150 °C and 10 Pa, including distillate 1 and distillate 2, as well as the residue are shown in Fig. 7. It can be noticed from Fig. 6 that the short path distillation is a useful technique for bio-oil fractionation to a certain degree at the condition applied. The distillate 1 fraction contained up to 51.3% fuel oil with 37.6% diesel and 10.6% kerosene, whereas the distillate 2 fraction contained mostly (53.14%) kerosene with 32% diesel and 14.4% fuel oil. The distillation residue fraction contained mainly (92.3%) fuel oil with small proportions of diesel (5.4%) and kerosene (1.5%). This demonstrates that the distillates 1 and 2 still need further purification or the conditions used in SPD may require optimization, which will be further investigated for our future study. Although fuel oil in residue fraction was very high, it could be improved by catalytic cracking; for example, heavy oil was upgraded by using TiO_2_ and ZrO_2_ catalysts in a fixed bed reactor and it can be converted to lighter fuel under superheated steam conditions [18]. In addition, the high heating value of the distilled fraction 1, fraction 2, and the stillage or residue were found to be relatively high at 37.51, 37.05, and 34.95 MJ/kg, respectively.

#### Lubricating properties

Bio-oil obtained from pyrolysis of oleaginous yeast was subjected to lubricity analysis. Three tribological results by the HFRR tests, consisting of wear scar diameter, film percentage, and friction coefficient are reported in Table 3. The mean wear scar diameter of ball specimen under the lubrication of bio-oil was 353.3 µm. This value is smaller than the acceptable limitation of wear scar diameter which is specified in the diesel fuel specification (ASTM D 975). Moreover, the wear scar diameter of bio-oil containing Oleic acid as main compositions is in an agreement of the previous publication which studied the lubricity of cis–trans isomers of C18:1 [49]. The mean value of 24.3% film concentration under the lubrication of bio-oil was obtained and the lubricating film was fluctuated among the tests. The low lubrication film concentration means that molecules of bio-oil are not easily absorbed on metallic surfaces to form strong and stable fluid film to prevent wear of moving parts. However, the friction coefficient generated during the test was quite stable, and the mean friction coefficient was 0.146.

**Table 3:** Results of HFRR lubricity test

Parameter	1st test	2nd Test	3rd Test	SD	AVG
Wear scar diameter (µm)	360	340	360	11.5	353.3
Film percentage (%)	20	33	20	7.5	24.3
Friction coefficient	0.147	0.145	0.147	0.00	0.146

**Fig. 8:**
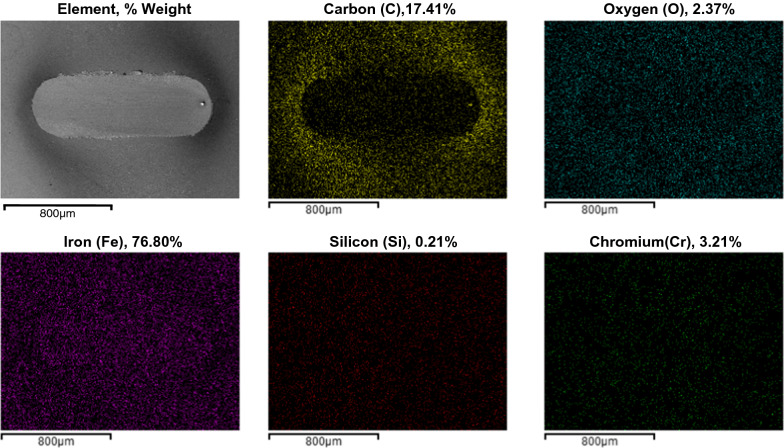
SEM images and EDX maps of wear scar on disk specimen

The microscopic analysis of the worn surfaces of disk specimens was further analyzed using the scanning electron microscope (SEM) equipped with energy-dispersive X-ray spectrometry (EDX). The SEM images and EDX maps are shown in Fig. 8. It was found that the residues of oxidation product were formed around the wear track of worm surfaces. The chemical compositions of the residues on the disk specimen under the lubrication of bio-oil were characterized. The EDX results show that almost all the black residue on the test disk was carbon. The presence of oxygen was also found as composition of the residues. The oxygen can be from the fuel molecules of bio-oil which possesses fatty acids as main components. Many studies reported that the presence of oxygen in fuel molecules can help to improve the lubricity of fuel [50, 51]. Other chemical compositions of worn surfaces were Fe, Si, and Cr, which are the components of the disk ANSI E-52100 steel specimens [52].

### Biochar characterization

**Fig. 9:**
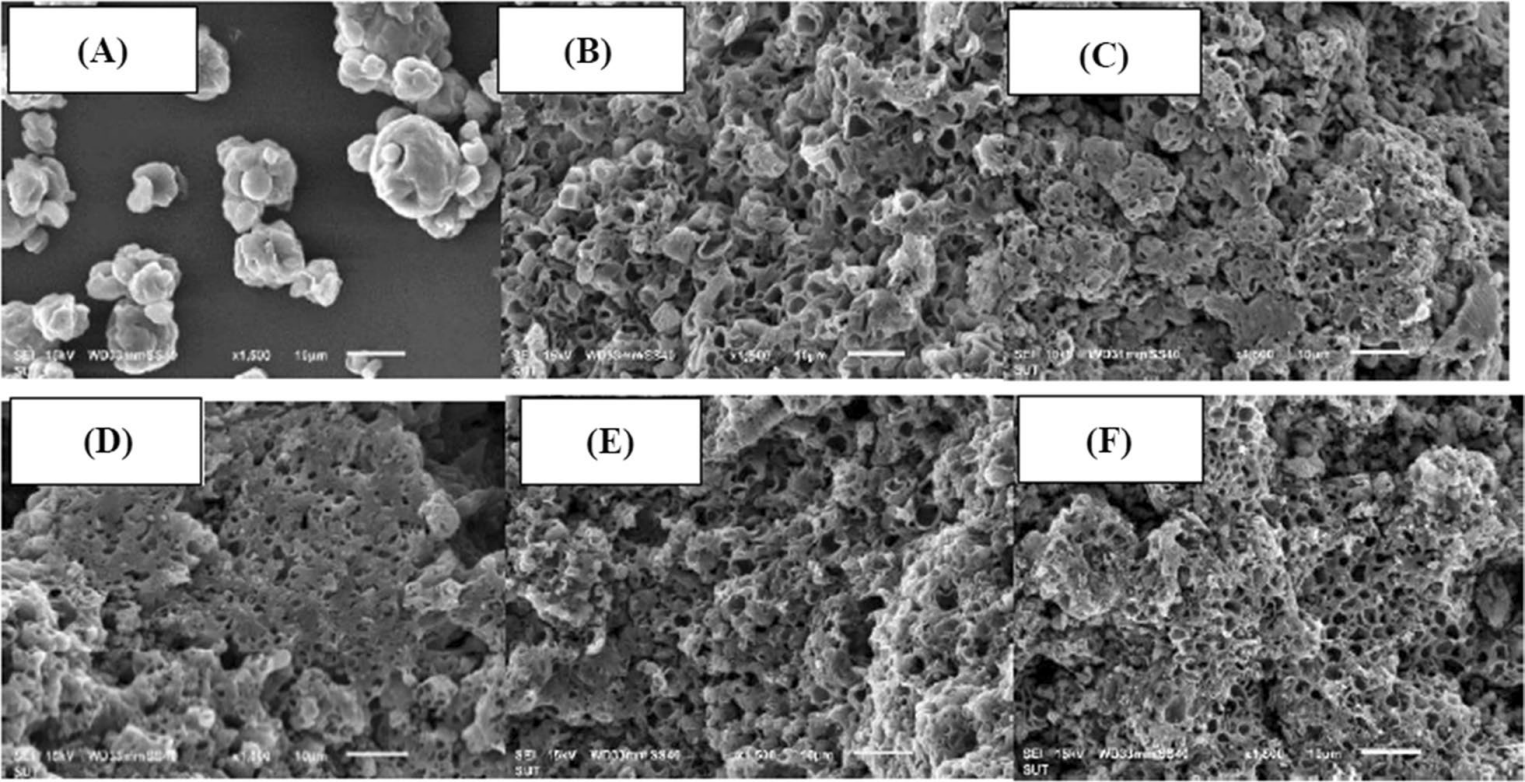
SEM images of **A** dried oleaginous yeast and biochar produced at **B** 400 °C; **C** 450 °C, **D** 500 °C, **E** 550 °C, and **F** 600 °C

**Fig. 10:**
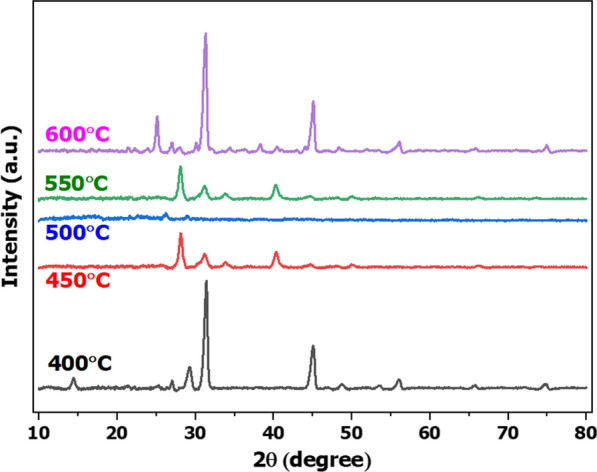
X-ray diffractogram (XRD) of biochar

**Fig. 11:**
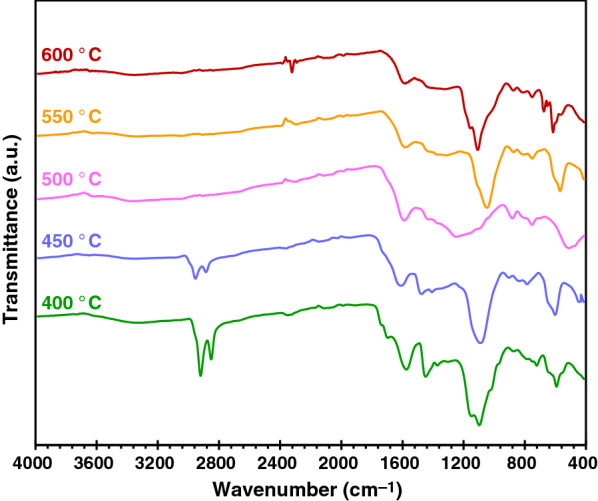
The FTIR spectrum of biochar

To gain an insight into the characteristics of biochar derived from oleaginous yeast, all biochar samples were tested for proximate analysis, ultimate analysis, heating value, surface properties, including BET surface area, pore size, pore volume and morphology by SEM, crystallographic structure by XRD and chemical functional group by FTIR, as shown in Table 4 and Figs. 9, 10, 11. It can be seen from Table 4 that the biochar was low in moisture content with only 2–5 wt%. In fact, the freshly produced biochar was bone dry (0% moisture) and in adsorbed moisture from the air during storage, indicating that biochar was somewhat hygroscopic. There is no apparent effect of pyrolysis temperature on biochar moisture content. For volatile and fixed carbon content, increasing the temperature from 400 to 550 °C led to the continuous decrease of volatile content from 51 to 17%. This occurs simultaneously with the increase of fixed carbon from 31 to 57%, indicating that the volatile evolved at elevated temperature until 550 °C. Further increase to 600 °C led to a sudden decrease of fixed carbon from 57 to 49%, thus causing the proportion of volatile slightly increases together with a sudden increase in the ash proportion from 21 to 27%. At the optimum pyrolysis temperature of 550 °C (based on the highest liquid bio-oil yield), the biochar contained approximately 21% ash with 57% fixed carbon. This biochar would be suitable as soil enhancer as if can slowly release fertilizer as well as capturing carbon in the ground, which can be regarded as a simple and nature carbon storage technology. When considering the elemental composition of the biochar samples, it was found that the biochar composed mainly of carbon with small proportions of oxygen, hydrogen, and nitrogen. The nitrogen may be in the form of proteins, which could potentially be extracted. The proteins may be extracted first before pyrolysis reaction. Table 4 also shows the biochar higher heating value, which was 22–29 MJ/kg compared to 23.2 MJ/kg for the raw yeast. It can be observed that increasing pyrolysis temperature led to the steady reduction of the biochar HHV.

**Table 4:** Properties of biochar derived from oleaginous yeast at different pyrolysis temperatures

Properties	Pyrolysis temperature (°C)				
Proximate analysis (wt%)	400	450	500	550	600
Moisture content	1.8	2.3	5.3	4.4	4.0
Volatile matter	51.1	33.7	22.9	17.3	19.6
Fixed carbon	31.1	47.0	53.2	57.0	49.1
Ash content	16.0	17.0	18.6	21.3	27.3
Ultimate analysis (wt%)					
Carbon	65.8	65.5	63.3	61.5	57.7
Hydrogen	6.5	5.6	3.5	3.1	2.6
Nitrogen	2.7	4.4	5.9	4.5	3.1
Oxygen	9.0	7.6	8.8	9.6	9.3
Heating value (MJ/kg)	28.8	27.9	27.7	24.3	21.8
Surface characteristics					
BET surface area (m^2^/g)	4.1	2.6	5.0	2.6	8.4
External surface (m^2^/g)	6.3	3.8	4.2	2.5	7.6
Total pore volume (mm^3^/g)	1.2	0.9	2.2	1.1	3.6
Average pore diameter (Å)	62.2	110.1	125.6	187.6	130.7

The comparison between HHV of biochar produced at 400–600 °C and standard coal demonstrated that HHVs of biochar were close to that of bituminous coal (27.267 MJ/kg)**.** The HHVs of other biochar (550–600 °C) and dried oleaginous yeast were close to those of coal or sub bituminous (23.97 MJ/kg). In theory, the bituminous included carbon content higher than sub bituminous. Consequently, the heating value of biochar was significantly increased with rising of carbon content.

The surface area of the yeast biochar was still very low compared to that of activated carbon. Therefore, further processing or activation is compulsory if the targeted application is related adsorption. Figure 9 shows the SEM images of the biochar in comparison with original yeast. It can be seen that the biochar had higher porosity than the raw material and increasing pyrolysis temperature led to slight increase of porosity. The biochar produced at the pyrolysis temperature of 600 °C appears to possess the highest surface area and porosity. This may be due to the more complete devolatilization of lipids and volatile matter present in the yeast sample. The effect of temperature on the surface characteristics of biochar derived from oleaginous yeast is rather different from other lignocellulosic materials. For example, durian wood (*D. zibethinus*) sawdust was used through a fixed bed reactor under an oxygen-free atmosphere at different pyrolysis temperatures (350, 450, and 550 °C). It was reported that the BET surface area increased from 2.57 to 221.0 m^2^/g when the pyrolysis temperature increased from 350 to 550 °C [12]. The BET results the specific surface area of the material increased from 4.74 to 462.83 m^2^/g with increasing pyrolysis temperature [29].

The XRD patterns for biochar are shown in Fig. 10. The structure and chemical compositions of biochar were depicted by identification of peaks from XRD. The strong peaks were found at range of 2θ = 25–45°. The XRD spectral analysis of biochar produced at 250–450 °C showed various peaks for example biochar at 250 °C, and found the strong peak at 2θ = 28.3°, 26.5°, and 31.4° showing the presence of fluorite, graphite, and chlorapatite. At 450 °C, the prominent sharp peak at 28.39° showed the presence of fluorite, while other high-intensity peaks at 31.41° and 40.36° were assigned to chlorite and gibbsite. In addition to these strong peaks, many other medium intensity peaks at 26.56°, 20.88°, and 45.34° (2θ) showed the presence of graphite, coquimbite, and hydrobiotite [53]. In this work, the strongest peaks of biochar produced at pyrolysis temperature of 400 and 600 °C were found at 2θ = 31.5°, possibly showing the presence of chlorapatite. The strongest peaks of biochar produced at the pyrolysis temperature of 450 and 550 °C determined at 2θ = 28.5° could be fluorite. Although peak of the biochar produced at the pyrolysis temperature of 500 °C disappeared, the other peaks were detected at 2θ = 26.5°, 41.0°, 45.0°, which cloud be possibly graphite, gibbsite, and hydrobiotite.

Biochar products were analyzed the functional groups by FTIR technique, the result shown in Fig. 11 with chemical functional groups given in Table 5. The peaks in the range of 592–646 cm^−1^ were assigned to alkane group (C–H). The peak around 675 cm^−1^ was due to the cis-disubstituted alkenes groups (C=C). The peaks in the ranges 721–752 cm^−1^ and 869–860 cm^−1^ were ascribed to the aromatic benzene (C–H). The peak around 1050–1150 cm^−1^ was due to the alcohol (C–O). The ethers group (C–O) was about 1100 cm^−1^. The peak at 1248 cm^−1^ might be ethers or carboxylic acid. The peak around 1380 cm^−1^ was assigned to methyl group (−CH_3_). The peaks in the range around 1450 cm^−1^ and 1580 cm^−1^ were ascribed as the aromatic ring. The peak at 2293–2322 cm^−1^ might be nitriles groups (C≡N). In addition, the results were reported that peaks at 2920 cm^−1^ and 2851 cm^−1^ were attributed to the presence of methylene group (CH_2_) [53]. According to Table 4 and Fig. 11, it is clear that biochar produced at the different pyrolysis temperature had partly similar chemical groups identified by the FTIR and also had certain different groups, such as methylene peak at 2851–2920 cm^−1^ which was found at low pyrolysis temperature (400–450 °C) and C≡N nitrile group which was identified at high pyrolysis temperature (550–600 °C).

**Table 5:** Functional groups of biochar samples determined by the FTIR Analysis

Specific type of bond	Wavenumber (cm^−1^) of biochar samples produced at different pyrolysis temperature (°C)				
	400	450	500	550	600
Alkane	592	567	551	569	–
Alkane	–	–	–	–	615
Alkane	–	–	–	–	646
Cis-disubstituted alkenes	–	–	–	–	675
C–H aromatic ortho-disub. benzene	721	750	752	752	751
C–H aromatic para-disub. benzene or trisubstituted alkenes	–	–	–	807	812
C–H aromatic meta-disub. benzene	873	869	880	873	874
C–O alcohol	–	1055	–	1046	
C–O esters	1097	–	–	–	1108
C–O alcohol	1149	–	–	–	–
C–O ethers, aromatic, or carboxylic acid	–	–	1248	–	–
C–H3 methyl	1372	1373	–	–	–
Aromatic C=C	1449	1440	–	–	
Aromatic C=C	1575	1575	1588	1580	1579
C≡N nitriles	–	–	–	2293	2322
Methylene	2851	2851	–	–	–
Methylene	2920	2919	–	–	–

## Conclusions

An oleaginous yeast *R. paludigena* CM33 could intracellularly accumulate 20–27% lipids depending of the carbon substrates. The lipids were composed mainly of oleic acid (C18:1) and palmitic acid (C16:0) with some other fatty acids. For the first time, this yeast strain has been pyrolyzed applying pyrolysis temperature in the range of 400–600 °C. The product distribution depended largely on the reaction temperature. At 550 °C, a maximum bio-oil yield of 60% could be achieved, whereas the highest biochar yield of 57.5% occurred at the lowest temperature of 400 °C. The produced bio-oil had higher heating value of 36 MJ/kg and contained mainly of C5–C18 compounds. According to the simulated distillation gas chromatography, the bio-oil contained 2.6% heavy naphtha, 20.7% kerosene, 24.3% biodiesel, and 52.4% fuel oil. Characterization of biochar revealed that it can be used as soil enhancer or solid fuel as the one produced at 550 °C consisted of 57% fixed carbon and 21% ash, with the heating value of 24 MJ/kg. The surface area of the yeast biochar was still very low compared to activated carbon. Therefore, if adsorption is a targeted application, the yeast biochar will definitely need a secondary processing.

## Data Availability

All data generated and analyzed in this study are included in this published article.
